# Living with uncertainty: associations of personal and environmental uncertainty, resilience, and self-efficacy with anxiety symptoms in young adults

**DOI:** 10.3389/fpubh.2026.1932199

**Published:** 2026-09-29

**Authors:** Annina Zysset, Matthew J Kerry, Julia Dratva

**Affiliations:** 1 Department of Health Science, Institute of Public Health, Zurich University of Applied Sciences, Winterthur, Switzerland; 2 Faculty of Medicine, University of Basel, Basel, Switzerland

**Keywords:** anxiety symptoms, collective efficacy, intolerance of uncertainty, polycrisis, public mental health, resilience, self-efficacy, young adults

## Abstract

**Background:**

Anxiety symptoms are highly prevalent among young adults and remain a major public mental health concern, with national data showing a marked increase during the COVID-19 pandemic. Contrary to expectations, anxiety rates did not decline after the pandemic ended. Persistent high anxiety levels may be contextually linked to an accumulation of concurrent global challenges, including climate change and geopolitical conflicts, that have been described as a “polycrisis” and are characterized by pervasive uncertainty. However, this framework was not directly examined in the present study. This study investigated the association between uncertainty, anxiety symptoms, resilience, and self-efficacy among university students.

**Methods:**

We used two datasets collected in Switzerland: a repeated cross-sectional pandemic study conducted during the COVID-19 pandemic (April 2020 to April 2023), and a post-pandemic cross-sectional study conducted in Fall 2023. First, general linear models were used to examine the trend of anxiety from Spring 2020–Fall 2023, combining data from both studies (N_wave_= 1,158). Second, using data from the post-pandemic study (*N* = 1,001), a hierarchical regression was conducted to examine the relationship between uncertainty, self-efficacy and resilience with anxiety symptoms, and to test for moderation effects of self-efficacy and resilience on the uncertainty–anxiety relationship.

**Results:**

A general linear model exhibited significantly better for a quadratic function compared to a linear function, suggesting an increase in anxiety followed by a saturation-plateau post-COVID-19 [β = −0.02, 95% CI (−0.03, −0.01)]. Regression results supported independent relationships [$R_{\left(\text{anxiety}\right)}^{2}$ = 39%], such that uncertainty positively was associated with anxiety [β = 0.50, CI (0.44, 0.56)], whereas self-efficacy [β = −0.22, 95% CI (−0.29, −0.15)], and resilience [β = −0.08, 95% CI (−0.29, −0.03)] were negatively associated. Moderation results were non-significant.

**Conclusion:**

Our findings highlight that uncertainty may play a role in sustaining high levels of anxiety among university students. Although high self-efficacy and resilience did not modify the relationship between uncertainty and anxiety symptoms, they were independently associated with lower anxiety levels. Addressing both global and specific facets of uncertainty appears to be highly relevant when designing targeted interventions to support mental health in young adults, particularly university students.

## 1 Introduction

Anxiety symptoms are highly prevalent among young adults (1–3). These disorders are defined as excessive and persistent worry or anxiety that significantly interferes with daily life (4). In the past years, studies have shown a marked increase in the prevalence of anxiety symptoms among young adults, a trend that accelerated during the COVID-19 pandemic (1, 5). The pandemic caused psychological distress due to sudden life disruptions, uncertainty, and isolation. While initial spikes were expected, post-pandemic data revealed persistently high rates of anxiety among young adults, suggesting underlying structural or environmental stressors (1, 6).

Young adults' susceptibility to anxiety may be rooted in emerging adulthood as a particularly vulnerable life phase. This life stage (18–25 years) is marked by developmental transitions and identity formation, which inherently increases vulnerability to psychological distress (7). Drawing on Havighurst's developmental tasks, this phase demands the acquisition of essential life skills, including decision-making related to personal goals, careers, and future planning. Due to neurodevelopmental changes and social role shifts, this phase is associated with a higher risk of mental health disorders (8).

In recent years, global crises have intensified the sense of uncertainty among young people, contributing to elevated psychological stress levels (9). The cumulative impact of global crises, such as the climate crisis, COVID-19 pandemic, and geopolitical conflicts, has created additional stressors for young people. Young adults report feeling overwhelmed by events beyond their control, contributing to uncertainty and distress (6, 9, 10).

In fact many stressors affecting young adults, whether personal (e.g., job insecurity, identity) or global (e.g., environmental crises), are characterized by pervasive uncertainty (11). Uncertainty is defined as the inability to predict outcomes or control events, which is a known risk factor for anxiety. The psychological theory of intolerance of uncertainty describes uncertainty as a key mechanism in mental disorders, such as depression and anxiety (12).

A growing body of evidence identifies uncertainty as a key psychological mechanism contributing to anxiety symptoms in youth, particularly after global disruptions (6, 12–14). A recent review stated that uncertainty might be a key driver of increased mental health problems in youth since the COVID-19 pandemic (15). For adolescents, who are supposed to be more flexible and less vulnerable to uncertainty, social uncertainty, such as isolation from friends during the pandemic, was stated to be a central problem for their mental health. Emerging adults today may be particularly vulnerable as they confront personal and growing global uncertainties (e.g., political instability, climate, and economic crises). These uncertainties are often intensified by constant exposure to media coverage, primarily through digital and social media, which amplify the perceived immediacy and severity of global crises (16, 17).

In addition to psychological mechanisms, sociodemographic characteristics influence exposure to uncertainty and the risk of developing anxiety symptoms (18). Socioeconomic factors play a crucial role in shaping how individuals perceive and respond to uncertainty, influencing their coping strategies and overall vulnerability to anxiety symptoms (19). Research consistently shows that women exhibit higher anxiety prevalence rates, which might be partly explained by a heightened perception of uncertainty (20), in addition to biological and other psychosocial factors (21). Similarly, individuals with a lower socioeconomic status or a migration background often face increased financial and social uncertainty due to structural disadvantages such as lower income, job instability, or limited access to resources. Previous studies have linked age, female sex (22, 23), LGBTQI+ identity (24–26), migration background (22, 27), and lower socioeconomic status (28) with a higher risk of anxiety symptoms. Given these established associations, these sociodemographic factors were included as covariates in the present study to control for their potential confounding effects when analyzing the relationship between uncertainty and anxiety.

The relationship between uncertainty, self-efficacy, resilience, and anxiety can be theoretically grounded in the transactional model of stress and coping (29), which conceptualizes psychological stress as arising from individuals' appraisal of environmental demands relative to their perceived coping resources. Within this framework, uncertainty may represent a source of perceived threat, whereas resilience and self-efficacy can be conceptualized as personal resources relevant to individuals' capacity to manage uncertain or stressful circumstances. This conceptualization is also consistent with contemporary models of uncertainty, which identify difficulty responding to uncertain or unknown circumstances as an important transdiagnostic process underlying anxiety and related emotional responses (12).

Beyond structural and demographic vulnerabilities, individual psychological resources, such as self-efficacy and resilience, may influence how uncertainty is experienced and managed among young adulthood. Self-efficacy and resilience are crucial in determining how well individuals cope with uncertainty-related stress. Self-efficacy refers to an individual's belief in their ability to manage challenges and exert control over their environment, fostering a greater sense of confidence and stability (30). Conversely, resilience enables individuals to adapt to adversity and recover more effectively from stress (31). Both factors are well-established protective mechanisms against the development of anxiety symptoms (32). However, while their general protective role is widely acknowledged, few studies have examined whether self-efficacy and resilience specifically moderate the relationship between uncertainty and anxiety among young adults. This study addresses the lack of research on the buffering effect of these protective factors on the uncertainty-anxiety relationship among emerging adults.

Building on previous findings, recent studies have highlighted the need to further investigate uncertainty as a key driver of persistently high anxiety levels among young adults. While uncertainty has been identified as a relevant factor in anxiety symptoms, its specific role in explaining sustained anxiety in the post-pandemic COVID-19 context remains underexplored, particularly given that young adults today face an accumulation of overlapping global crises beyond COVID-19, including geopolitical conflicts, economic instability, and climate crisis (33). Furthermore, little is known about which specific uncertainty domains are most salient for this population. Research suggests that protective factors, such as resilience and self-efficacy, should be examined in the context of uncertainty-related anxiety, yet their moderating role in this context has rarely been tested. The present study first, investigates the continued anxiety levels in both the pandemic study and the post-pandemic study data by testing whether anxiety symptoms followed a non-linear pattern over the course of the COVID-19 pandemic and beyond. Second, it addresses these gaps by testing three hypotheses: (1) Perceived uncertainty is associated with higher anxiety symptoms among young adults in a Swiss student sample; (2) Self-efficacy and resilience are associated with lower anxiety symptoms; and (3) Self-efficacy and resilience moderate the positive relationship between uncertainty and anxiety, such the higher levels weaken the relationship.

## 2 Materials and methods

### 2.1 Study design and study sample

The data used in the current study were collected in two studies: “Health in Students during the Corona pandemic (HES-C)” (22, 34, 35), and “Health in Students at Zürich University of Applied Sciences (HES-Z)” (36).

HES-C is a repeated cross-sectional survey conducted during the COVID-19 pandemic (April 2020 to April 2023) among students at the Zurich University of Applied Sciences (ZHAW), a university of applied sciences in the German-speaking part of Switzerland. Students of the ZHAW, approximately 12′430 at each wave, received non-personalized emails inviting them to participate in the studies. The average sample size per wave was at *n* = 1,158. HES-Z followed the same methodology as HES-C, using an online survey in Germin among ZHAW students, but was conducted only once, in Fall 2023 (*N* = 1,001). The repeated cross-sectional design aligns with similar studies examining anxiety trajectories surrounding the COVID-19 pandemic (37). Repeated cross-sections is also appropriate given our theoretical transactional model conceptualizes stress responses as a dynamic process over time (29).

To examine the trajectory of anxiety symptoms during and after the pandemic, data from both study datasets were combined: HES-C as the pandemic study, and HES-Z as “post-pandemic study,” containing a single post-pandemic measurement point. Hypotheses regarding uncertainty, self-efficacy and resilience and anxiety were investigated using data from the post-pandemic study (HES-Z) only. Both studies were approved by the local ethic committee (BASEC-Nr. Req.-2020-00326, BASEC-Nr. Req-2023-01117).

### 2.2 Measures

All measures used in the present study have demonstrated adequate psychometric properties in student populations and have been applied in recent COVID-19 and post-pandemic research, supporting their continued relevance in the current context.

#### 2.2.1 Uncertainty

Uncertainty was measured with the uncertainty subscale from the Stress—und Coping-Inventar (SCI), developed by Satow (38) which assesses which assesses uncertainty across seven life domains, including finances, housing, work or studies, family, relationships, health, and personal goals, on a scale from 1 to 7, with higher values indicating greater perceived uncertainty. For the present study, one additional item assessing global uncertainty (e.g., geopolitical conflicts, climate crisis) was added to the subscale to better capture the current uncertainty landscape faced by young adults. An exploratory factor analysis supported a unidimensional factor structure for the adapted subscale, justifying its use as a composite score. Domain-specific scores were not analyzed separately, as the unidimensional factor structure did not support the treatment of individual uncertainty domains as distinct constructs.

The original instrument development also reported factor analytic eigenvalue ratios approaching 3, suggesting essential unidimensionality from using all factors. The SCI uncertainty subscale has been applied in recent studies examining stress and uncertainty in student populations during and after the COVID-19 pandemic (39). Reliability, indexed by McDonald‘s omega (ω) coefficient was estimated at ω = 0.73 in the current study.

#### 2.2.2 Anxiety

Anxiety was measured with the *General Anxiety symptom-Scale-7* (GAD-7), developed by Spitzer et al. (40) and validated in the German-speaking population by Löwe et al. (41). The GAD-7 is a self-assessment questionnaire that measures the level of perceived anxiety in the last 2 weeks. The questionnaire comprises seven items to be rated on a 4-point Likert scale (from “not at all” to “nearly every day”). The resulting sum score ranges from 0 to 21, with higher values indicating greater anxiety severity. A cut-off score of ≥ 10 indicates moderate-to-severe anxiety symptoms, in line with established thresholds for clinically relevant anxiety. The GAD-7 is one of the most widely used screening instruments for anxiety symptoms in population-based and epidemiological research, including studies conducted during and after the COVID-19 pandemic (41–43). In the current study, reliability was estimated at ω = 0.89.

#### 2.2.3 Self-efficacy

The General Self-efficacy Expectations Short Scale (ASKU), developed by Beierlein et al. (44), based on the General Self-Efficacy Scale by Jerusalem and Schwarzer (45). The ASKU records individual expectations of competence in dealing with difficulties and obstacles in daily life. Therefore, three statements had to be answered on a 5-point Likert scale (1 = doesn't apply at all, 5 = applies completely). Total score ranges from 3 to 15. The ASKU has been used often in the past 10 years, also in studies in students during COVID-19 pandemic (22, 46). Reliability was estimated at ω = 0.83 in the current study.

#### 2.2.4 Resilience

Resilient coping was measured using the Brief Resilient Coping Scale (BRCS), developed by Sinclair and Wallston (47). This instrument is based on four questions, which are answered on a 5-point Likert scale (1 = doesn't describe me at all, 5 = describes me very well), with total scores ranging from 4 to 20. The measure addresses a central protective factor associated with concept of wellbeing (47) and has been used in various studies also recently during the COVID-19 pandemic, including cross-cultural validation studies examining resilient coping in student populations (48, 49). The scale has a reliability of ω = 0.611 in the current study.

#### 2.2.5 Sociodemographic variables

Collected were age (year of birth), gender identification (male, female, diverse), sexual orientation (heterosexual vs. non-heterosexual), nationality (Swiss vs. Non-Swiss nationality), subjective parental social status at student age 16 years (McArthur Scale). These variables were included as covariates based on prior research indicating their significant association with anxiety prevalence and their potential confounding effects in the uncertainty-anxiety relationship.

### 2.3 Statistical analyses

Descriptive statistics (i.e., frequencies, percent, mean, and standard deviation) were applied to describe the characteristics of the sample. Age- and sex-adjusted anxiety levels were calculated to compare anxiety prevalence in our student sample from data collected during the COVID-19 pandemic (Spring 2020–Fall 2023). *P-values* < 0.05 are interpreted for significance. Multivariate linear regression analyses were performed to investigate the association between demographic, uncertainty, and anxiety, as well as hypothesized protective factors (self-efficacy, resilience). The model was developed hierarchically including sets of co-variates consecutively. In the first step (Model 1), sociodemographic variables were entered to determine the extent to which they were associated with the outcome. In the second step (Model 2), we added uncertainty, self-efficacy and resilience to examine whether they explained additional variance in anxiety symptoms beyond the sociodemographic variables and their independent association with the outcome. The third step was to investigate moderating effects between these co-variates, possibly adding to the variance of anxiety explained by the model. Predictors were entered hierarchically from relatively distal and generic to increasingly proximal and condition-specific factors: demographic characteristics, socioeconomic characteristics, generic health-related variables, and condition-specific variables. The order of entry was specified *a priori* based on theoretical and empirical considerations and the principle of causal priority in hierarchical regression (50).

The GAD-7 sum score was analyzed as a continuous outcome in the temporal trajectory and regression analyses. The GAD-7 ≥10 threshold was used only descriptively to characterize the proportion of participants screening positive for clinically relevant anxiety symptoms and was not treated as a clinical diagnosis. Hierarchical regressions tested for moderation effects by entering interaction terms into prediction equations. Exploratory factor analysis (EFA) using principal axis factoring was conducted to examine the dimensionality of the uncertainty scale and its suitability for use as a general predictor in the regression model. Sampling adequacy was evaluated using the Kaiser–Meyer–Olkin (KMO) measure and Bartlett's test of sphericity. Varimax rotation was implemented. The number of factors was evaluated based on the scree plot, 1st/2nd eigenvalue rations, and interpretability of the factor solution. Statistical analyses were conducted using IBM SPSS Statistics for Windows, Version 31.0 (IBM Corp., Armonk, NY, USA) and R version 4.6.0 (R Core Team, 2026).

## 3 Results

Less-than 5% of our total sample exhibited partial-missing data. Missingness ranged from 0.1%−1.8% across all variables. Given the low magnitude (< 5%), complete-case analysis proceeded. This was supported by non-significant Little missing-completely-at-random (MCAR) tests, ranging from *p* = 0.11–93.

Regression assumptions were evaluated prior to analysis. Multicollinearity was assessed using variance inflation factors (VIFs), which ranged from 1.12 to 1.90 and indicated no evidence of problematic multicollinearity. Residual diagnostics indicated some departure from normality and evidence of heteroscedasticity; therefore, heteroscedasticity-consistent robust standard errors were used for statistical inference (51). Examination of standardized residuals and influence diagnostics indicated few extreme observations (*n* < 3) and no cases with Cook's distance approaching 1 (52). Linearity was additionally assessed using residual diagnostics, and independence of observations was ensured by the study design. Our EFA for justifying the use of uncertainty as a general predictor exhibited a Kaiser–Meyer–Olkin (KMO) measure of sampling adequacy of 0.814, and Bartlett's test of sphericity was significant, χ^2^(36) = 1,039.26, *p* < 0.001. The scree plot indicated a pronounced dominant first factor (first eigenvalue = 2.88; second eigenvalue = 1.03; ratio = 2.80), providing support for an essentially unidimensional representation of the scale.

### 3.1 Sample characteristics and descriptives statistics

Descriptive results of the post-pandemic study sample characteristics are shown in Table 1. The highest uncertainties were reported for global events (*M* = 3.3, *SD* = 1.9), the personal domains, financial problems (*M* = 3.5, *SD* = 2.0), and important life goals (*M* = 3.2, *SD* = 1.7). Lowest uncertainty for unspecified/other (*M* = 1.9, *SD* = 1.6). Descriptive analyses showed that GAD-7 mean score was 7.9 (*SD* = 4.7) and 27.3% were in the GAD-7 category minimal, 39.1% in mild, 22.1% in moderate, and 11.5% in the category severe anxiety symptoms. Overall, 33.6% of students scored ≥10 on the GAD-7, indicating clinically relevant anxiety symptoms according to the established screening threshold.

**Table 1:** Study sample descriptive characteristics.

Variable	*n*	%	Mean/sum ±sd
Gender identity			
Women	712	71.4	
Men	263	26.4	
Diverse	22	2.2	
Age (years)	1,001		25.0 ± 6.5
Social status (score 1 to 10)	982		5.6 ± 1.9
Sexual orientation			
Heterosexual	804	81.5	
Non-heterosexual	183	18.5	
Swiss nationality			
Swiss nationality	900	89.9	
Non-swiss nationatily	101	10.1	
Anxiety (sumcore)	991		7.9 ± 4.7
Uncertainty (possible range: 1 to 7)	817		2.7 ± 1.1
Self-efficacy (possible range: 1 to 5)	999		3.8 ± 0.6
Resilience (possible range: 4 to 20)	998		15.2 ± 2.2

### 3.2 Trajectory of anxiety from Spring 2020–Fall 2023

The trajectory of age- and sex-adjusted mean GAD-7 scores from Spring 2020 to Fall 2023 is shown in Figure 1. Estimates represent marginal means derived from the general linear model. Our data show that anxiety symptoms increased during the COVID-19 pandemic (wave 1–9), and it remained elevated at the single Fall 2023 post-pandemic assessment (Figure 1).

**Figure 1:**
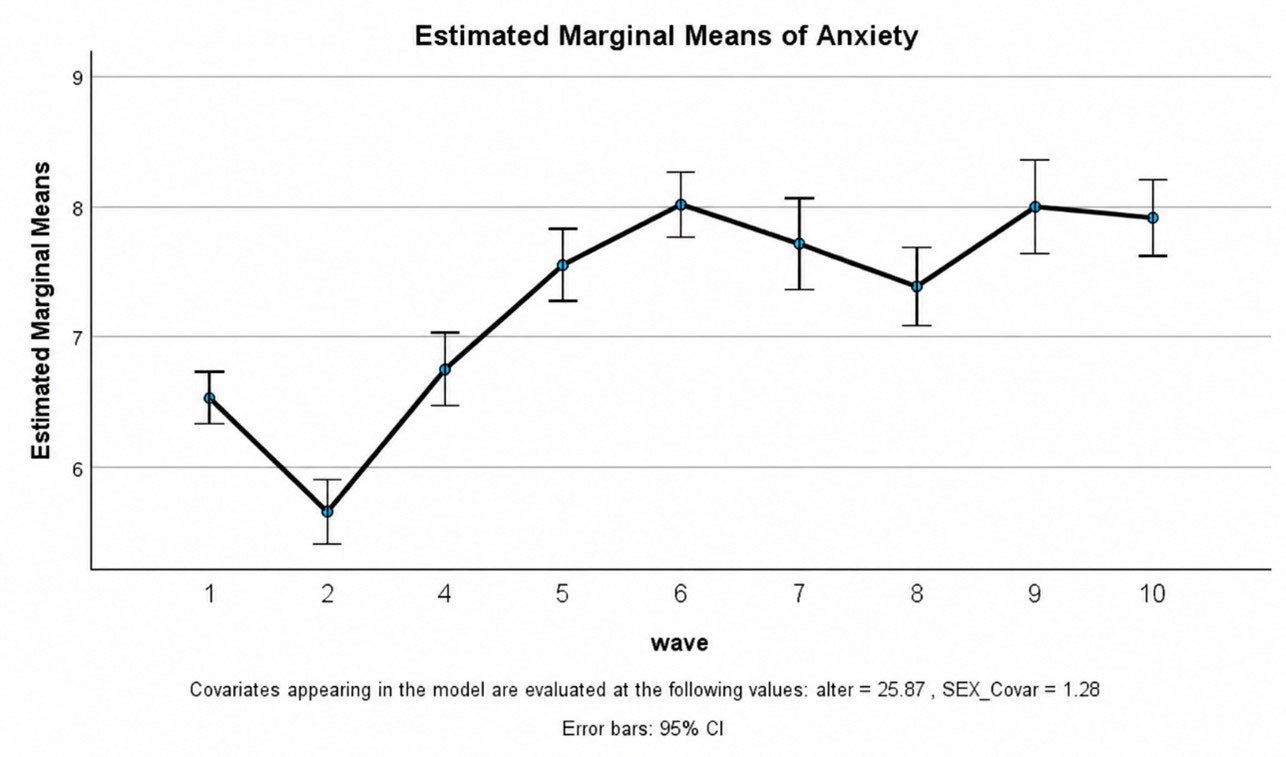
Adjusted prevalences of generalized anxiety symptoms from Spring 2020 to Fall 2023.

In fact, our first approach, the estimated baseline-linear model exhibited significance, *F*_(3,10,420)_ = 87.84, *p* < 0.001, with wave positively predicting anxiety [β = 0.214, SE = 0.015, *t* = 13.99, 95% CI (0.185, 0.243)]. Next, we added wave^2^ to test for non-linearity. The quadratic term exhibited statistical significance [β = −0.017, SE = 0.006, *t* = −2.90, 95% CI (−0.029, −0.005)], indicating a plateauing of anxiety scores toward the end of the pandemic. Model comparison showed that the quadratic model provided a significantly better fit than the baseline linear model, *F*_(1,10,419)_ = 8.42, *p* = 0.0037. Information criteria also favored the quadratic specification [Akaike Information Criterion (AIC) = 61,975.9] over the linear model (AIC = 61,982.3), with a ΔAIC of 6.4.

### 3.3 Associations between uncertainty, self-efficacy and resilience, and anxiety

Results for all three Models are reported in Table 2. In the first step (Model 1), sociodemographic variables were entered to determine the extent to which they were associated with anxiety symptoms (GAD-7 sum score). Younger age was related to higher anxiety, *B* = −0.06, SE = 0.03, β = −0.08, *p* = 0.022. Sexual orientation was significantly associated with anxiety, with non-heterosexual participants reporting higher anxiety scores than heterosexual participants, *B* = 1.20, SE = 0.44, β = 0.10, *p* = 0.007. Self-identified gender showed a small effect, with women reporting higher anxiety than men, *B* = 0.80, SE = 0.38, β = 0.08, *p* = 0.039, whereas participants identifying with another gender did not differ significantly from men, *B* = 1.39, SE = 1.25, β = 0.04, *p* = 0.268. Lower socioeconomic status was associated with higher anxiety, *B* = −0.22, SE = 0.10, β = −0.08, *p* = 0.020. Nationality (Swiss vs. non-Swiss) was unrelated to anxiety, *B* = −0.02, SE = 0.55, β = −0.001, *p* = 0.968. Model 1 explained 3.1% of the variance in GAD-7 sum score (*R*^2^ = 0.031, adjusted *R*^2^ = 0.024).

**Table 2:** Hierarchical regression predicting anxiety symptoms (GAD-7 sum score).

Predictors	Model 1				Model 2				Model 3			
	*B*	SE	[95% CI]	β	*B*	SE	[95% CI]	β	*B*	SE	[95% CI]	β
Age (years)	−0.06	0.03	[−0.11, −0.01]	**−0.08**	−0.04	0.02	[−0.08, 0.00]	−0.05	−0.04	0.02	[−0.08, 0.00]	−0.05
Gender: female vs. male	0.80	0.38	[0.04, 1.55]	**0.08**	0.06	0.31	[−0.55, 0.67]	0.01	0.06	0.31	[−0.55, 0.67]	0.01
Gender: diverse vs. male	1.39	1.25	[−1.07, 3.84]	0.04	0.55	1.00	[−1.42, 2.51]	0.55	0.57	1.00	[−1.39, 2.54]	0.02
Sexual orientation: non-heterosexual vs. heterosexual	1.20	0.44	[0.33, 2.07]	**0.10**	0.01	0.36	[−0.69, 0.72]	0.00	0.00	0.36	[−0.71, 0.71]	0.00
Nationality: CH vs. non-CH	−0.02	0.55	[−1.11, 1.06]	0.00	0.85	0.44	[−0.02, 1.72]	0.06	0.86	0.44	[−0.01, 1.73]	0.06
Socioeconomic status	−0.22	0.10	[−0.41, −0.03]	–**0.08**	0.09	0.08	[−0.06, 0.24]	0.03	0.10	0.08	[−0.06, 0.25]	0.04
Uncertainty (MC)					2.35	0.14	[2.06, 2.63]	**0.50**	2.35	0.14	[2.07, 2.63]	**0.50**
Self-efficacy (MC)					−1.61	0.24	[−2.09, −1.13]	–**0.22**	−1.63	0.25	[−2.11, −1.15]	–**0.22**
Resilience (MC)					−0.16	0.07	[−0.29, −0.03]	–**0.08**	−0.16	0.07	[−0.29, −0.03]	–**0.08**
Uncertainty ^*^ Self-efficacy									0.17	0.22	[−0.27, 0.60]	0.02
Uncertainty ^*^ Resilience									0.02	0.06	[−0.10, 0.15]	0.01

*N* = 778. Unstandardized coefficients (B) with standard errors (SE) are reported, along with 95% confidence intervals, standardized coefficients (β), and significance levels (*p*-values). MC = mean-centered. Betas *p* < 0.05 = bold. ^*^mathematical multiplication/product terms between the two variables comprising the computed interaction term.

In the second step (Model 2), uncertainty, self-efficacy, and resilience were added. Higher uncertainty was strongly associated with higher anxiety scores, *B* = 2.35, SE = 0.14, β = 0.50, *p* < 0.001. Lower self-efficacy was also related to higher anxiety, *B* = −1.61, SE = 0.24, β = −0.22, *p* < 0.001, as was lower resilience, *B* = −0.16, SE = 0.07, β = −0.08, *p* = 0.017. None of the sociodemographic variables remained significant in Model 2. Model 2 explained 38.4% variance in GAD-7 sum core (*R*^2^= 0.384, adjusted *R*^2^ = 0.376), substantially improving model fit compared to Model 1.

In the third step (Model 3), two interaction terms were added. The interaction term uncertainty × self-efficacy (*B* = 0.17, SE = 0.22, β = 0.02, *p* = 0.450) as well as the interaction term uncertainty × resilience (*B* = 0.02, SE = 0.06, β = 0.01, *p* = 0.744) were not significant. Model 3 explained 38.5% of the variance in GAD-7 sum core (*R*^2^ = 0.385, adjusted *R*^2^= 0.376). Thus, model 3 added no more explained variance.

## 4 Discussion

The present findings confirm that anxiety symptoms in university students increased during the COVID-19 pandemic, indicating a plateauing of anxiety scores toward the end of the pandemic and sustained high level of anxiety in Fall 2023 rather than a return to pre-pandemic levels. Regarding the three hypotheses, the findings support Hypothesis 1: higher perceived uncertainty was significantly associated with higher anxiety symptoms. Hypothesis 2 was also supported: higher self-efficacy and resilience were significantly associated with lower anxiety symptoms. Hypothesis 3 was not supported: self-efficacy and resilience did not significantly moderate the relationship between uncertainty and anxiety. With respect to Hypothesis 1, descriptive analyses revealed that the three uncertainty domains with the highest perceived burden in the student sample were financial concerns, personal life goals, and global events.

Addressing the research question on the temporal trajectory of anxiety, the present study examined whether anxiety symptoms in young adults in a student population followed a non-linear pattern over the course of the COVID-19 pandemic and beyond. We expected the trend of increasing anxiety in the pandemic to plateau rather than continually increase. A nested-model comparison indicated that a quadratic specification provided a statistically better fit than a linear model, although the increase in practical predictive power was small. This suggests that while anxiety levels did not continue to rise indefinitely, they also did not meaningfully decline—consistent with a plateau rather than a recovery. This extends existing evidence, which has predominantly shown that anxiety levels remained elevated post-pandemic, by showing a non-linear trajectory consistent with a leveling-off of anxiety scores toward the end of the observation period. This finding is in line with national and international studies reporting similarly sustained, elevated anxiety levels beyond the acute phase of the pandemic (1, 53).

**Hypothesis 1** stated that perceived uncertainty is associated with higher anxiety symptoms among university students. We observed a significant positive correlation, as well as a significant regression coefficient incremental to demographics in predicting anxiety. This finding supports our first hypothesis, and it is consistent with international research (14). The present findings are consistent with research positioning uncertainty as a transdiagnostic risk factor for anxiety symptoms (12, 14). Specifically, intolerance of uncertainty describes the tendency to react negatively to uncertain situations and has been shown to amplify anxiety across a range of clinical presentations. Importantly, longitudinal evidence supports a prospective role of intolerance of uncertainty in anxiety: Adamis et al. (13) found that intolerance of uncertainty in 2016 significantly predicted coronavirus anxiety and COVID stress syndrome in 2020, with worry functioning as an additional interactive vulnerability factor, such that individuals with both high intolerance of uncertainty and high trait worry experienced the most severe anxiety symptoms. These findings suggest that intolerance of uncertainty is not merely a correlate of anxiety, but an underlying vulnerability that shapes responses to large-scale uncertainty events—a mechanism directly relevant to the persistent anxiety rates observed in the present study. Ingrassia et al. (54) further demonstrated that intolerance of uncertainty negatively predicted wellbeing among young adults across Armenian and Italian contexts, while future time perspective served as a positive predictor. This suggests that the role of uncertainty extends beyond anxiety symptoms to broader indicators of psychological health, and may operate differently across cultural settings. It is important to note, however, that the current study only measured perceived uncertainty, which is substantively distinct, although related to, uncertainty intolerance.

Prior research further suggests that the psychological impact of uncertainty depends on its perceived proximity. According to Construal Level Theory (55), distal and abstract threats, such as climate crisis or geopolitical conflicts, are processed at greater psychological distance and are therefore expected to have a weaker direct effect on anxiety than proximal stressors such as financial strain or personal life goals. Financial uncertainty has indeed been identified as a strong predictor of anxiety and depression among young adults in the study by Liu et al. (56). Ogunbode et al. (57) found that the association between climate-related anxiety and mental health outcomes became non-significant when loneliness and financial anxiety were considered in the model, consistent with the view that proximal, concrete stressors exert a more immediate psychological impact than distal threats (55, 58). However, Regnoli et al. (59) found that the fear of war significantly predicted stress, anxiety, and depression among young Italian adults, partly through increased intolerance of uncertainty and future anxiety, suggesting that even geographically distant conflicts can become psychologically proximal, particularly when mediated through intolerance of uncertainty. Longitudinal evidence further supports the relevance of such distal stressors: concerns about wars, economic crises, and climate crisis increased significantly among young people in Germany from 2020 to 2024, alongside persistently impaired mental health (53).

The present findings suggest that distal stressors may be similarly salient to proximal ones, at least in terms of perceived relevance. Global events ranked descriptively among the top three uncertainty domains, in terms of perceived burden, alongside financial concerns and personal life goals. This pattern may be explained by the increased use of digital and social media. Real-time coverage of geopolitical conflicts, climate disasters, and public health threats through platforms such as Instagram, YouTube, and TikTok, renders these events more immediate and personally relevant than traditional media did, potentially reducing the psychological distance typically associated with global stressors. This may help explain why global events were as salient as more proximal uncertainties in the present sample and raises the question of whether the traditional distinction between proximal and distal uncertainty remains meaningful for a generation with continuous, real-time access to global crises.

**Hypothesis 2** stated that self-efficacy and resilience are associated with lower anxiety symptoms. We observed a significant negative correlation, as well as a significant regression coefficient for both constructs, indicating that higher self-efficacy and resilience were independently associated with lower anxiety symptoms incremental to demographics in predicting anxiety. This finding supports our second hypothesis and is consistent with existing research demonstrating the protective role of these resources against anxiety and psychological distress (32).

The observed associations are theoretically consistent with the transactional model of stress and coping (29), which conceptualizes self-efficacy and resilience as personal coping resources that moderate individuals' appraisal of stressors. Specifically, self-efficacy may reduce anxiety by fostering a sense of competence and agency in the face of uncertainty (30). Resilience, in turn, enables individuals to adapt to adversity and recover more effectively from stressful experiences (47), potentially attenuating the emotional impact of perceived uncertainty over time.

These findings are particularly relevant in the post-pandemic context, where young adults face an accumulation of concurrent stressors. Even under conditions of heightened and persistent uncertainty, self-efficacy, and resilience appear to exert a consistent protective effect on anxiety—suggesting that strengthening these resources remains a meaningful intervention target, even when they do not buffer the uncertainty–anxiety relationship directly (see Hypothesis 3).

**Hypothesis 3** stated that self-efficacy and resilience moderate the positive relationship between uncertainty and anxiety, such that higher levels weaken the relationship. A hierarchical regression analysis tested the interaction terms. We observed non-significant relationships (60). The null finding for Hypothesis 3 may be interpreted in two ways, with different implications. First, a moderation effect may exist in the broader population but remained undetected in the present study due to restricted variance in resilience and self-efficacy scores. Self-efficacy and resilience scores in the present sample were relatively high, approaching the upper end of their respective scales (see Table 1), suggesting a ceiling effect consistent with a positively selected student population. This restricted range in protective factor scores could have reduced statistical power to detect moderation effects (60). Under this interpretation, strengthening resilience and self-efficacy in more heterogeneous populations could still prove a useful intervention approach, and future research with broader samples is warranted. Second, and alternatively, moderation may genuinely not exist: self-efficacy and resilience may exert the same effect on anxiety regardless of the level of perceived uncertainty. Under this interpretation, raising resilience is not a sufficient intervention strategy for reducing the impact of uncertainty on anxiety—a conclusion that motivates the conceptual argument developed below. In light of our null findings, a *post-hoc* power analysis with *N* = 778 and α = 0.05 with observed effect = 16% power. To achieve 80% power, a sample size of approximately *n* = 6,000 participants would have been needed to detect our observed effects. Also, a sensitivity analysis indicated that Δ*R*^2^ = 0.008 would have been the smallest effect we could have detected with our current sample size.

One possible theoretical interpretation of this pattern draws on what has been termed a polycrisis—the simultaneous and mutually reinforcing occurrence of multiple large-scale stressors (61). The current generation of young adults has faced a series of overlapping collective crises: the COVID-19 pandemic, geopolitical conflicts, and climate crisis. These crises do not operate in isolation; they compound one another and accumulate over time, potentially shifting uncertainty from a temporary stressor to a more structural feature of early adulthood. Under such conditions, individual-level coping resources may be insufficient not because they are ineffective *per se*, but because some stressors are simply too large to be managed at the individual level. This interpretation is further supported by the nature of the uncertainty domains most salient in the present sample. As discussed, global events were among the uncertainty domains perceived as most burdensome alongside financial concerns and personal life goals—domains that largely reflect stressors outside individual control. Self-efficacy theory posits that perceived control is central to the efficacy-wellbeing link (30): individuals feel more capable and less anxious when they believe their actions can influence outcomes. When the dominant stressors are structural and collectively determined, individual agency has limited purchase, which may help explain why self-efficacy and resilience, though beneficial, did not buffer the uncertainty–anxiety relationship in the present study.

These considerations suggest that collective efficacy may represent a theoretically relevant construct worthy of future investigation. Collective efficacy refers to the shared belief that meaningful change is achievable through coordinated action (62), as a potentially more powerful protective construct in the context of global uncertainty. Unlike individual self-efficacy, collective efficacy locates agency at the group or societal level, which may be better matched to the structural nature of the stressors young adults currently face. In the context of climate anxiety, collective engagement and a sense of shared agency have been proposed as more effective than individual resilience alone (63, 64). While the present study did not measure collective efficacy directly, these theoretical considerations suggest it represents a promising and empirically underexplored avenue for future research and intervention that warrants investigation in the context of broader global uncertainty, beyond climate anxiety alone. Resilience is not absent or ineffective—it demonstrably lowers anxiety. But when structural uncertainty becomes a permanent condition rather than a temporary disruption, the question shifts from how do we make individuals more resilient to resilient to what, and can this remain an individual achievement?

### 4.1 Limitations

First, the cross-sectional design of the study precludes the establishment of causal relationships between uncertainty, protective factors, and anxiety. While our findings indicate significant associations, longitudinal studies are needed to determine the directionality of these effects. Second, the study sample comprised university students from a single institution in Switzerland, which may limit the generalizability of our findings to broader young adult populations or other cultural contexts. Additionally, voluntary participation via non-personalized emails might have resulted in a self-selected sample, raising concerns about potential selection bias (e.g., students experiencing impaired health or mental health might have been more inclined to participate in the student health survey). Third, uncertainty was analyzed as a composite score in the regression model, precluding conclusions about the differential impact of specific uncertainty domains, such as financial, global, or life goal related uncertainties on anxiety outcomes. Furthermore, the present study focuses specifically on perceived uncertainty as a predictor of anxiety and did not account for other established risk factors such as loneliness and perfectionism. Prior research has identified these as significant contributors to anxiety and depression among young adults (5, 57). The coping scale exhibited a relatively lower reliability, which should be further examined in future research. We must also note that the sample was characterized by a gender imbalance, with women representing 72% of participants. This distribution is typical of allied health profession students, but nonetheless limiting the generalizability of findings to young men. This imbalance may have reduced the statistical power to detect gender-specific effects and may have influenced the regression estimates, given that women consistently report higher anxiety prevalence rates than men. Although gender was included as a covariate in all regression models to partially control for this imbalance, future studies should examine the robustness of these findings in more gender-balanced samples. We also have to mention that, while repeat-participation was possible, anonymized surveys prohibited the use of individual-level identifiers and, therefore, clustering-based corrections to models. Furthermore, the trajectory analysis is based on a repeated cross-sectional rather than a longitudinal design. As a result, the observed changes in anxiety over time may partly reflect differences in sample composition across waves rather than true population-level change. Although age and sex were included as covariates in all analyses, other relevant characteristics may have varied across waves. This represents an inherent limitation of repeated cross-sectional designs and should be considered when interpreting the temporal findings. Additionally, the absence of a pre-pandemic baseline measurement limits the ability to establish whether anxiety levels have returned to, remained above, or continued to change from pre-pandemic levels. The Fall 2023 assessment represents a single post-pandemic measurement point and should not be interpreted as evidence of a stable post-pandemic trend.

### 4.2 Future research

Several directions for future research emerge from the present findings. First, longitudinal studies are needed to establish the directionality of the relationship between perceived uncertainty and anxiety, and to capture the cumulative psychological burden of repeated crisis exposure. Second, future studies should examine specific uncertainty domains, financial, global, and life-goal-related, as separate predictors, to determine which exert the strongest influence on anxiety outcomes and whether their effects differ across subgroups. Third, the role of social media consumption warrants investigation as a potential moderator between global uncertainty exposure and anxiety, particularly whether frequent crisis coverage intensifies the psychological impact of distal stressors by increasing their perceived proximity. Fourth, collective efficacy should be examined as a protective factor against uncertainty-related anxiety. Finally, future studies should extend beyond student samples to broader young adult populations and across cultural contexts, to improve generalizability. Furthermore, with a true repeated-measures dataset, future research incorporating mediation or moderated mediation models would allow a more comprehensive examination of the pathways through which uncertainty influences anxiety, building on the transactional model of stress and coping.

### 4.3 Conclusion and implications for public health

The present study confirms that perceived uncertainty is significantly associated with anxiety symptoms among university students, consistent with the hypothesis that persistent post-pandemic anxiety may be contextually linked to an accumulation of concurrent structural stressors. Resilience and self-efficacy were independently associated with lower anxiety, but no evidence of moderation was detected, a finding that may point to the limits of individual coping resources in the context of a polycrisis. Together, these results suggest that effective public health responses to anxiety in university students and young adults more broadly may benefit from addressing both individual resources and structural conditions producing uncertainty.

The present findings have implications for public health practice at two levels. Where possible, implications are directly derived from the present findings; additional recommendations draw on the broader intervention literature and should be understood as theoretically informed directions rather than conclusions directly supported by the present data. At the population level, interventions should be matched to the type of uncertainty involved. For proximal stressors such as financial concerns or life goal uncertainty, cognitive-behavioral approaches targeting perceived uncertainty represent an evidence-based option, supported by the broader intervention literature on intolerance of uncertainty (65). For global stressors, fostering collective efficacy may represent a promising complementary approach, though this was not directly examined in the present study, with preliminary support from the climate anxiety literature (63, 64). More broadly, public health responses should address the structural conditions producing uncertainty, not only their psychological consequences at the individual level. This includes, for example, integrating media literacy education into curricula to reduce the psychological impact of continuous crisis exposure, or by ensuring that financial support structures for university students and young adults more broadly are framed explicitly as mental health interventions.

Universities are particularly well-positioned to reach young adults during a critical developmental period. Drawing on the present findings and the broader intervention literature, three priorities for the university setting can be identified. First, psychoeducation about uncertainty and its psychological effects represent a low-threshold intervention that could be integrated into existing student health services or delivered digitally. A review of reviews found evidence that mindfulness-based interventions, Cognitive Behavioral Therapy (CBT), and technology-delivered interventions were effective for student mental health when compared to a passive control, with CBT-based interventions showing sustained effects over time (66). Digital delivery is particularly relevant given capacity constraints in university counseling services. Second, financial concerns were descriptively among the most salient uncertainty domains in the present sample, suggesting the potential relevance of practical structural support such as financial counseling, emergency funds, and student housing support. Third, for global stressors, universities can foster collective engagement through student societies, volunteering, and participatory formats. Peer support is associated with reductions in depression, loneliness, and anxiety among university students, with lower barriers to access compared to traditional mental health services (67), and peer mentoring and peer learning have shown positive results for anxiety and stress in higher education settings specifically (68). Involving students in the design of mental health initiatives through participatory workshops and student-led peer platforms has been shown to further improve acceptability and reduce stigma (69). Taken together, an effective university mental health strategy should operate across these levels simultaneously.

## Data Availability

The raw data supporting the conclusions of this article will be made available by the authors, without undue reservation.
